# Human Growth and Body Weight Dynamics: An Integrative Systems Model

**DOI:** 10.1371/journal.pone.0114609

**Published:** 2014-12-05

**Authors:** Hazhir Rahmandad

**Affiliations:** 1 Industrial and Systems Engineering Department, Virginia Tech, Falls Church, Virginia, United States of America; 2 Sloan School of Management, Massachusetts Institute of Technology, Cambridge, Massachusetts, United States of America; McMaster University, Canada

## Abstract

Quantifying human weight and height dynamics due to growth, aging, and energy balance can inform clinical practice and policy analysis. This paper presents the first mechanism-based model spanning full individual life and capturing changes in body weight, composition and height. Integrating previous empirical and modeling findings and validated against several additional empirical studies, the model replicates key trends in human growth including A) Changes in energy requirements from birth to old ages. B) Short and long-term dynamics of body weight and composition. C) Stunted growth with chronic malnutrition and potential for catch up growth. From obesity policy analysis to treating malnutrition and tracking growth trajectories, the model can address diverse policy questions. For example I find that even without further rise in obesity, the gap between healthy and actual Body Mass Indexes (BMIs) has embedded, for different population groups, a surplus of 14%–24% in energy intake which will be a source of significant inertia in obesity trends. In another analysis, energy deficit percentage needed to reduce BMI by one unit is found to be relatively constant across ages. Accompanying documented and freely available simulation model facilitates diverse applications customized to different sub-populations.

## Introduction

The obesity trends across the world are alarming [1], [2] and span different age, gender, and ethnic groups [3] with significant health and economic costs [4]. Validated computational models are needed to assess the impact of alternative interventions and policies. For example use of simplistic models can lead to overly optimistic expectations of an intervention that may later disappoint and hurt adherence [5]. Computational models of weight and height dynamics can also inform monitoring of individual growth and aging, design and evaluation of malnourishment and stunted growth interventions, and diagnosis of eating and growth disorders, among others. Finally, mathematical models integrate existing findings, provide quantitative predictions, and motivate empirical studies that target model-identified knowledge gaps.

A growing literature includes models of body weight dynamics in adults [6]–[10]. A few such models exist for childhood growth [11], [12]. However, current models do not 1) Include infants and children younger than five years of age. 2) Capture dynamics of growth in height. 3) Consider longer-term dynamics including changes in body composition due to aging. 4) Capture racial differences or provide clear points to customize the model for specific sub-populations. This paper introduces a mechanistic model of individual body weight, composition, and height dynamics from birth to old ages, including variations across individuals with respect to gender and race. The model is validated using a diverse set of prior studies not used in model formulation, enhancing robustness and applicability to intervention design and policy analysis. Documented simulation model and instructions are provided to enable replication, extensions, and diverse analyses by interested researchers and practitioners.

## Materials and Methods

A mechanistic modeling approach is pursued in which a system of ordinary differential equations represents human body with state variables representing key concepts needed to quantify body weight, height, and composition through life. I use three state variables for this purpose. Following previous research, body weight is partitioned into fat mass (FM) and fat free mass (FFM). The latter combines in a single variable protein, glycogen, intra and extra-cellular fluid masses, and other non-fat components. FM and FFM suffice for modeling longer-term dynamics discussed in this paper [10], while more detail is required for capturing hourly and daily dynamics [9]. A third state variable, height (H), allows this model to account for variations in height and the potential for stunted growth as a result of malnutrition. Inclusion of height also facilitates the use of BMI, rather than weight, in specifying reference inputs for the model and analyzing obesity and other conditions. There is less variation in BMI than there is in weight, making the resulting reference curves more robust.

Besides modeling height endogenously, two guiding ideas distinguish the current model from the previous research and enable new features. Below I discuss how the model is formulated based on these two ideas: the canalization of growth and the allocation of energy. Detailed model formulations and original models with instructions for running the models are available in the supporting files (Appendices S1, S3, and S4), here the key processes are summarized.

At the heart of the model is the idea that human growth is canalized through childhood [13], therefore there is a natural tendency to close the gap between current values and indicated ones for H, and to some extent FM, and FFM. I use this idea to specify the formulations for changes in different state variables (H, FM, and FFM). Specifically, to capture height growth during childhood, deviations from indicated height, 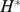, signals the desired height velocity for normal growth. This desired growth rate is then modified based on the availability of energy needed for growth, thus actual height is guided by H*, but at times may fall behind that. The indicated height for an individual aggregates many complex genetic and environmental determinants below the model's level of aggregation into a single curve. I use the reference 50^th^ percentile Centers for Disease Control and Prevention (CDC) growth charts for specifying H* as a function of age [14], and modify that for three race effects based on height differences observed in National Health and Nutrition Examination Survey (NHANES) data [15]. In this study I use the data from continuous NHANES waves between 1999 and 2008 and any reference to NHANES data refers to this subset.

The model captures changes in weight and body composition by allocating the available energy supplied (i.e. energy intake (EI)) to various demands in the body (i.e. for basic metabolism and repair of existing tissue, as well as deposition of new tissue in the growth phase). The imbalance between the supply and demand will lead to deposition of FM and FFM (if supply exceeds demands) or slow-down of growth and loss of existing mass (if demand exceeds supply). The demands for energy to be allocated to FM and FFM growth are determined by their indicated values, which at any point in time inform the natural growth trajectory for a simulated individual. I assume a balanced diet and therefore do not disaggregate the energy intake to consider the composition of macronutrients in the diet.

Indicated body weight (BW*) is calculated based on the indicated height and the indicated body mass index (BMI*). The latter is formulated as a weighted sum of the current body mass index (*BMI*), and a biological reference body mass index (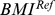), specified by reference CDC growth charts [14]. This formulation accounts for the more limited canalization of BMI, compared to height: whereas height is rather tightly regulated by its genetic determinants and thus a fixed curve sets its indicated values, reference BMI is partially dependent on the current BMI. The indicated BW* is partitioned into indicated FM and FFM (FM* and FFM*) based on an empirical equation (Equation 1). This equation, estimated using NHANES data for subjects between 8 and 50 years of age (for whom body composition measures were available in two rounds of the survey), calculates the most likely Fat Mass Index (*FMI*) for any given BMI value. It bases its estimates on reference values of FMI (*FMI^Ref^*) from Wells, Butte, and colleagues [16], [17], and modifies those based on individual's BMI status (*BMI^Ref^-BMI*) and race. Looking at deviations from reference BMI allows one to separate the variations in composition due to growth and aging, from those due to weight gain and loss, and thus offers more precise predictions. Impact of age on body composition after childhood is also incorporated in reference FMI curve using a linear function (estimated on NHANES data). A comparison with the most commonly used alternative partitioning equation, the Forbes equation [18], suggests that this formulation is potentially more precise (See Appendix S2 for details).
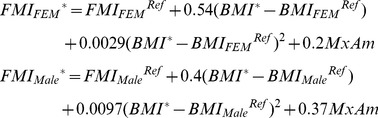
(1)


FM and FFM change rates will follow the indicated trajectory if body composition is on the indicated trajectory and energy that is demanded for normal growth and aging is matched by energy supplied. In the absence of energy balance or deviations from indicated composition, an allocation process determines which sources of energy contribute to what mechanisms' demand for energy. If energy demanded for growth and maintenance exceeds energy intake, energy will be supplied from metabolizing FM and FFM stores in the body. On the other hand if EI exceeds demand, stores of FM and FFM act as a reservoir, balancing the total energy supply and demand. Furthermore, any deviation from the composition indicated by the current BMI (which can be calculated using equation 1) is corrected through changing supply and demand of energy from FM and FFM. This mechanism captures the homeostatic processes that keep the body composition on a regulated path [18]. Therefore FM and FFM can both supply and demand energy while EI is always a source and basal metabolic rate (BMR), physical activity (PA), and turnover of mass are always demanding energy. Energy balance is enforced by assigning sources of energy to demands based on priorities of supply and demand: EI is used completely before extra FM and FFM (masses beyond reference trajectory) are tapped into for energy supply, and only when these sources are exhausted essential FM and FFM (masses composing the reference trajectory) may be utilized for survival. On the demand side maintenance needs are first satisfied, followed by deposition of essential FM and FFM, and only then extra EI may deposit FM or FFM. Deposition (and consumption) of extra mass into FM vs. FFM follows a partitioning equation derived from the trajectory indicated by current BMI (equation 1). Specifically, the following partitioning equation (Equation 2) results if FMI takes the functional form we use, i.e. 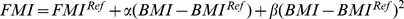:
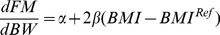
(2)


The metabolizable energy content of FM is assumed constant and a linear relationship describes FFM energy content as a function of FFM [12], saturating at an adult value of 5 MJ/kg.

Equation 3 summarizes the maintenance and growth components of demand for energy (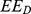). Energy demand for BMR is calculated based on the energy demands of liver, brain, heart, kidney, and the remaining FM and FFM, as they change with age based on data from Altman and Dittmer [19]. These values are adjusted for the relative cellularity of organs from birth and through life according to Wang [20]. The relative sizes of these organs change as individual's weight deviates from the indicated values, changing the BMR energy demand based on estimates by Hall [9]. Energy costs of FM and FFM storage and turnover are calculated based on indicated mass velocities. (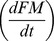 and 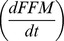). 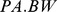 calculates the energy need for physical activity, where reference PA values are taken from Torun's estimates [21] for childhood and adjusted during adulthood based on trends in NHANES data. The metabolic cost of feeding is captured in the term 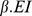. Finally, the ratio of EI and the energy demand for the previous components, when applied to BMR (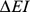 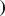, is used to calculate changes in total energy expenditure due to adaptive thermogenesis [22]. The individual will follow the reference growth trajectory if 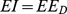 and deviations in EI from this value lead to weight change based on the energy allocation mechanism.
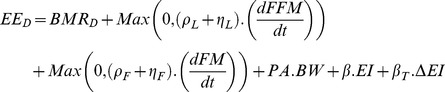
(3)


Reliable data on the impact of energy deficit on slowing height growth is not available due to long time horizons over which these dynamics unfold and the ethical and measurement challenges. Nevertheless the current model is parameterized to reflect three stylized findings regarding stunting and catch up growth [23]. First, children who, due to malnutrition, have fallen behind their growth trajectory, show rapid catch up growth when provided with adequate nutrition, up to four times as fast as their age-specific normal growth rate [24]. Second, growth in height is stopped by adulthood, and in the absence of enough catch up growth before that stunted children may never reach their potential height. Finally, the growth in height will be hampered if the individual is under 85% of their normal BMI for age [25].

The model allows for capturing variations across individuals and subpopulations at multiple levels. First, indicated values could be adjusted for race. I use the NHANES data to estimate population level adjustments to H* for Mexican-American, none-Hispanic white, and none-Hispanic black. I also include race as an independent variable in the FMI* regressions (see equation 1). Second, calendar age and biological age (used in model equations) are separated, so that biological age is an increasing function of calendar age. Thus slow (fast) growing children could be simulated by a constant multiplier smaller (bigger) than one. Nonlinear mappings can represent more complex growth patterns but are harder to estimate empirically. Third, a parameter reflecting individual genetic variations in potential height adjusts the H* around population level reference values. Similar parameters are incorporated to capture variations in predisposition to higher BMI (than reference), more fat mass than average (after controlling for BMI and Race), and physical activity. Given the focus of this paper, unless explicitly specified, I do not use these individual level adjustments in the remainder of the paper and show individual trajectories consistent with gender and race specific averages for H*, BMI*, FMI*, and PA. Simulation analysis is conducted using Vensim software, with Euler integration time step of one day (results are not sensitive to smaller values for time step). Statistical analysis is conducted using Stata software.

## Results

The current model provides the first mechanistic model of growth and weight dynamics that spans birth to old ages and incorporates height dynamics. Three steps are included in the analysis. First, model predictions are compared against previous empirical studies not used in the construction of the model. These validation simulations allow for building confidence in the model and identifying potential discrepancies. Given that no prior model exists for infancy and early childhood weight dynamics as well as height dynamics across ages, comparison with empirical samples is required for understanding the model's strengths and weaknesses. Next, for variables and age ranges where estimates from previous models can be obtained, the new model is compared with predictions from those alternatives and sources of variation are discussed. This discussion provides additional insights into the differences in mechanisms and modeling assumptions across existing model architectures. Those insights can guide future modelers and inform experiments needed to tease out alternative formulations. Finally, questions relevant to obesity and growth are explored in a few example analyses.

Table 1 reports on multiple comparisons of model simulations against empirical studies where weight, height, composition, or energy expenditure components are available. Unless specified a) base parameters for a reference individual are used in producing representative simulations that are compared to empirical sample measures; b) simulations start at birth and continue to the age at which empirical samples are collected. The observed differences between model and empirical results are discussed next. First, the model predicts lower BMR and TEE for a sample of 9-month old male Swedish infants, though the TEE discrepancy could be partially explained by higher PA levels in the reported sample than the model. Also, higher-than observed prediction for the weight of the female sample from this study at 14-month of age leads to higher BMR predictions [26]. Similarly, taller-than-reference empirical samples result in lower weights and FFM (though statistically significant in only one of the four comparisons) than observed in a study of young adults [27]. Moreover, two potential deviations are observed in estimating BMR. First, the model underestimates BMR for two of the childhood samples [28], [29]. This may indicate a general bias in the model, but may also be partly explained by different measurement protocols from the basal levels formulated in simulations. For example Salbe and colleagues measured REE for obese children after 10 minutes of rest [28], which is likely to produce larger REE values compared to basal levels. The other difference is in over-estimating (though not statistically significant) BMR for a sample of elderly subjects [27]. The model captures a reduction in BMR with age due to a shift in body composition with aging towards having more fat mass, and a reduction in cellular density with age [30], and is consistent with another sample of elderly [31]. Nevertheless, the observed discrepancy may indicate a more significant dependence of BMR components on aging than is captured in the model and warrants further study into how components of BMR change with age.

**Table 1:** Comparison of model projections against multiple samples.

n(Gender)	Age	Weight (kg)	FFM (kg)	FM (kg)	Total Energy Expenditure (MJ/Day)	Resting Energy Expenditure (MJ/Day)	Height	Source
30(M)	9 m	9.74 (0.88)		2.92 (0.62)	3.12 (0.25)	2.19 (0.18)	*0.74 (0.01)*	[26] *
		9.91		2.46	**2.85**	**1.99**	*0.74*	
14(F)	9 m	9.33 (0.95)		2.8 (0.72)	2.99 (0.26)	1.99 (0.17)	*0.73 (0.02)*	
		9.98		2.62	2.82	2.00	*0.73*	
17(M)	14 m	11.34 (1.52)		3.24 (0.84)	3.72 (0.28)	2.52 (0.32)	0.8 (0.03)	
		11.54		2.79	**3.29**	2.28	0.80	
12(F)	14 m	10.24 (0.81)		3 (0.61)	3.19 (0.22)	2.15 (0.14)	0.79 (0.02)	
		11.58		2.83	3.31	**2.31**	0.79	
65(M) &73(F)	5	*22.8 (5)*	*15.8 (2.2)*		5.97 (0.91)	4.39 (0.5)	1.14 (0.05)	[28] †
		*22.79*	*15.81*		5.47	**3.50**	1.12	
65(M) &73(F)	10	*51.8 (15.2)*	30.4 (5.4)		9.54 (1.35)	6.2 (1.06)	1.46 (0.07)	
		*51.80*	30.46		10.07	5.40	1.43	
28(F)	9.9 (0.4)		25.3 (3)	8.4 (2.5)	8.17 (1.36)	5.22 (0.67)	1.41 (0.06)	[29]#
			24.26	8.72	7.63	**4.54**	1.40	
28(F)	11.9 (0.4)		32.3 (4.4)	13.1 (4.7)	9.35 (1.39)	5.93 (0.69)	1.54 (0.07)	
			31.09	11.64	9.28	5.32	1.54	
24(F)	14.8 (0.4)		42 (4.4)	16.4 (4.2)	10.36 (1.32)	5.85 (0.65)	1.65 (0.06)	
			38.30	15.16	10.34	5.91	1.63	
21(F)	16.6 (0.9)		43.4 (4.4)	17.2 (4.6)		5.82 (0.62)	1.67 (0.06)	
			42.27	18.39		6.03	1.64	
15(M)	11.5 (1.8)	*65.3 (18.2)*	39 (10)	26.3 (10.1)			1.51 (0.09)	[32]&
		*65.48*	43.81	21.67			1.49	
15(M)	11.6 (1.9)	40.1 (12.1)	32.4 (11.4)	7.7 (3.6)			1.49 (0.16)	
		39.35	31.65	7.71			1.50	
23(F)	11.2 (2.2)	*74.9 (26.6)*	40.2 (11.6)	34.6 (16)			1.52 (0.11)	
		*75.18*	43.03	32.15			1.48	
23(F)	11.3 (2.2)	39.7 (10.2)	30 (7.6)	9.6 (3.9)			1.47 (0.15)	
		38.71	28.30	10.41			1.48	
27(F)	23 (2)	63.4 (6)	45.6 (3.2)			5.88 (0.48)	1.71 (0.05)	[27]
		58.64	**41.42**			5.72	1.64	
29(M)	27 (2)	77.2 (9.1)	66.4 (7)			7.62 (0.76)	1.85 (0.07)	
		72.90	59.71			7.35	1.78	
164(F)	67.7 (5.6)	67.5 (9.7)	37.2 (4.7)			5.5 (0.65)	1.595 (0.055)	[31]
		58.64	41.05			5.32	**1.64**	
98(M)	67.1 (5.2)	78.6 (9.3)	53.2 (5.3)			6.79 (0.75)	1.732 (0.065)	
		72.90	56.97			6.50	**1.78**	
71(F)	75 (5)	66.9 (9.4)	40.8 (4.4)			4.8 (0.56)	1.61 (0.06)	[27]
		58.64	40.99			5.26	1.64	
32(M)	73 (6)	78 (11.1)	54.2 (6.3)			5.73 (0.66)	1.76 (0.06)	
		72.90	56.58			6.38	1.78	

Empirical results include mean and standard deviation (in parenthesis) of each outcome, and below that the simulation model's projections. Unless specified, simulation model results report baseline projections with no attempt to fit the data. Simulation numbers are made bold if they fall outside of mean +/-standard deviation. Where model parameters were fitted to match a specific outcome, the comparison for that outcome is made italic.

* Infant's individual height factor is fitted to start from 9 month old sample means.

† Model fitted to initial weight, height, and fat fraction. Energy intake provided to match final weight. Simulation starts at age 5.

# Energy intake provided to match the final weight.

& Energy intake provided to match final weights for overweight samples (65.3 kg for male and 74.9 for female).

Overall, in the large majority of comparisons (79 out of 89) the simulated measures fall within one standard deviation of empirical sample averages, and few systematic errors are detected across a wide range of ages and different subpopulations. The model's predictions closely match body composition and energy expenditure not only among normal samples, but also among large infants [26], overweight [29] and obese children [28], [32], and elderly [27], [31]. Body composition deviations from empirical samples are small and do not show any consistent directional bias, providing further confidence in the projections produced by the model.

**Figure 1:**
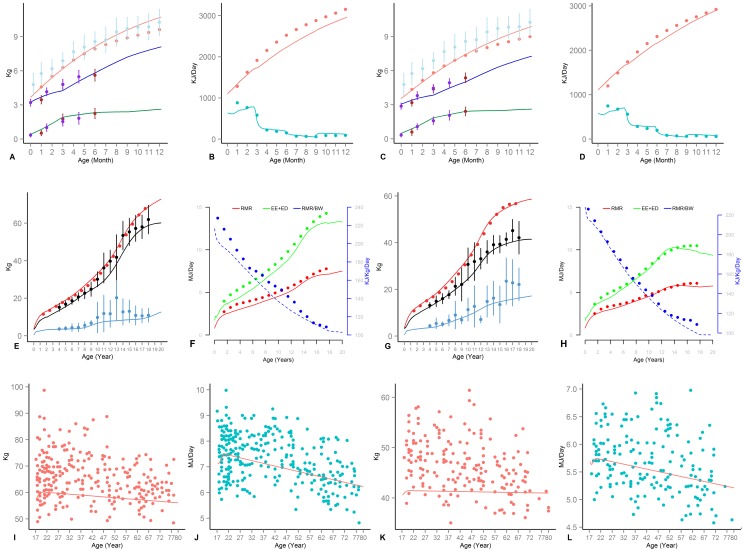
Comparison of model (solid lines) and infant (Panels A–D), child (Panels E–H), and adult (Panels I–L) empirical results. Error bars represent one standard deviation for empirical samples. (A & C) Male and Female; BW compared to [35] (light red) and NHANES non-hispanic white (blue); FFM (higher) and FM (lower) compared to [33] (purple) and [34] (red). (B & D) Male and Female; energy expenditure (red) and Growth energy needs (blue) compared to [35] (E & G) Male and Female; BW compared to [36] (red); FM (blue) and FFM (black) compared to [37]. (F & H) Male and Female; Total Energy Requirement (Green) and BMR (Red) compared to [36] (left Y-axis); BMR/BW compared to [38] (blue; right Y-axis). (I& K) FFM compared with samples from [39] and [40] for male and female. (J & L) BMR compared with samples from [39] and [40] for male and female.

Comparisons between model simulations and longitudinal data and reference curves not used in model estimation are presented next. In Figure 1, model predictions (solid lines) are compared with data from longitudinal studies of male (two columns on the left) and female (the two columns on the right) subjects among infants (Panels A–D), children (Panels E–H), and adults (Panels I–J). Infant growth is compared with data on FM, FFM [33], [34], reference body weight [15], [35], and energy requirements (for growth and total energy needs) [35]. The difference between the model projections for infants and various empirical samples is similar to the differences among different samples. The energy requirements for growth show very close correspondence to the reference data, yet male total energy expenditure in the model is slightly below the Butte's reference (Panel B), partly because the physical activity reference curves used in the model have a low resolution and do not capture variations over the first months of life. Childhood (Panels E–H) weight simulations correspond very closely to Torun's reference [36], and FM and FFM values are in concert with results reported by Ellis and colleagues [37], though slightly under-estimating fat mass for males between 11–15 years of age (Panel E). Across both genders and over childhood years energy expenditure values are also consistent for BMR, total energy expenditure (including growth; compared against Torun's reference [36]), and BMR per unit of weight (compared against Talbot reference value [38]); model predictions are slightly below these reference values for male (Panels F). Finally, comparisons of FFM (Panels I and K) and BMR (Panels J and L) over adulthood with cross sectional data on male [39] and female [40] subjects are consistent in magnitude and trends. The higher FFM values in empirical subjects compared to the reference simulated subject is expected given the prevalence of obesity and the fact that we compare the data against simulations of a normal subject. Declining trend in empirical FFM data likely combines changes in obesity profiles across different age groups, as well as a declining trend in FFM fraction due to aging. The parallel decline in the simulated trends only captures the latter source of decline and is less pronounced.

**Figure 2:**
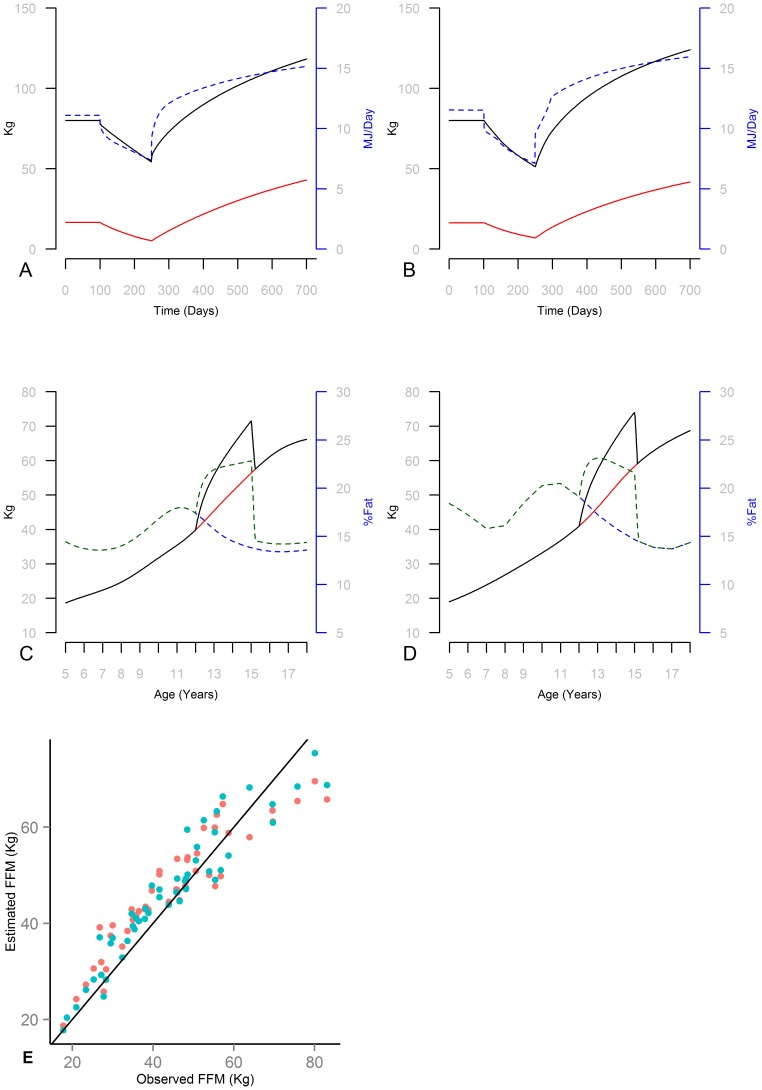
Comparison of current model with existing models. (A & B) H1 and current model simulating a 30-year-old, 80 kg, white male subject switching to 1500 Kcal/Day below initial (equilibrium) diet on day 100 and then to 1500 Kcal/Day over initial on day 250; FM (red) and BW (black solid, left axis) and energy expenditure (dashed blue; right axis) are graphed. (C & D) H2 and current model simulating a male child from age 5 to 18 in reference growth and with 20% more EI than reference between ages 12 and 15 with later compensating EI to get back to reference growth curve; BW under reference and extra energy (Red and Black, solid lines, left axis) and Fat Percentage (Blue and Green dashed lines, right axis). (E) FFM predicted by H2 (Red) and Current model (Blue) against observed values for 50 NHANES subjects.

Comparisons between model predictions and those from other computational models are summarized in Figure 2. In panels A and B results from the current model (Panel B) are compared with the behavior of an extensively validated model for adults [10] (H1; in Panel A). H1 predictions are generated using the online body weight simulator provided by the National Institute of Diabetes and Digestive and Kidney Diseases. Panels A and B show H1 and the current models' projections for FM (red), BW (black), and TEE (dashed blue, right axis) for a simulated 30 year old male subject. Starting from equilibrium weight of 80 kg, he first switches to a reduced calorie diet 1500 kcal/day below initial EI at day 100, and then on day 250 switches to an overfeeding condition with 1500 kcal/day over the initial energy intake, which is pursued for another 450 days. This extreme scenario provides a test of models' robustness with significant under/over feeding cases. Starting (equilibrium) energy expenditure is similar across the H1 and the current model: 11.1 vs. 11.5 MJ/Day respectively. The initial body composition is almost identical (20.7% vs. 20.5% fat fraction for H1 and current model). Overall trends are also similar across the two models with two minor variations. Energy expenditure curves from H1 are smoother because of the differences in formulating adaptive thermogenesis (AT). H1 includes a separate state variable for AT and the current model takes into consideration the energy need for catch up growth in estimating the energy gap that leads to AT. Moreover, the body composition equation in the current model (equation 2) leads to slightly slower change in the composition of added/lost body mass compared to the Forbes partitioning equation [18], [41] (used in H1) in extreme cases. This difference induces slightly faster weight gain and loss under extreme conditions in the current model. Some statistical analysis (see Appendix S2) supported the use of the current functional form for estimating indicated FMI. Nevertheless the variations in weight change across H1 and current model are small even in these extreme conditions: minimum weights (at time 250) are 54.3 kg vs. 51.4 kg and final weights are 118.3 Kg vs. 124.1 kg respectively.

Panels C and D report on a comparison between the one other empirically validated model of childhood weight gain and loss [12] (H2; in Panel C), against the current model (Panel D). In this experiment a male subject during his normal growth years (starting at 5 (earliest age in H2) until 18 years of age) is exposed to an increased energy diet offering 120% of his normal energy intake between ages 12 and 15 years old (reference EI used before that). Next deficit intake is imposed until the simulated subject reaches his normal-for-age weight, at which point the reference intake is reinstated. Results for reference vs. experimental condition for weight (red and black solid lines) as well fat fraction (blue and green dashed lines; right axis) are shown. The models show similar overall patterns with two differences. First, current model predicts slightly more weight gain (maximum weight gain of 15.7 vs. 15.2 in H2). This is largely due to marginally higher reference weight (and thus energy intake) in the base case in the current model. The models also differ in the formulation of AT, which in H2 is based on the variation of EI from a reference individual, but in the current model depends on variation from maintenance energy needs for the individual at the current weight. Differences in body composition trajectories are also notable, resulting from different partitioning and allocation equations used. In the current model a single energy partitioning equation is estimated for adults and children, while H2 has modified Forbes equation (which is based on adult data) to match childhood trajectories. While both models match the reference body composition well, more comparisons with empirical body composition among obese or underweight children can inform the relative precision of the two models further from the reference curves.

The more substantive difference is in the behavior of fat fraction after the weight returns to the reference curve. In the current model once the individual returns to the normal weight for age curve, his fat fraction also goes back to the reference curve. The H2 model however shows a steady-state rise in the fat fraction despite the weight recovery. A similar steady-state difference (a drop in fat fraction) is observed when a malnutrition condition is simulated (not shown; note that H2 is not designed for simulating malnutrition). This difference points to a key distinction between the two models. The current model controls weight dynamics to represent two separate mechanisms: first, it adjusts body composition to track the indicated FMI for the current BMI; second, it deposits or consumes mass due to energy gap based on a partitioning equation. The other models in the literature (including H1 and H2) focus on the latter mechanism, and adjust for changes in body composition over age (in case of H2) by modifying the energy partitioning equation with an age dependent term during childhood. This mechanism offers a simpler mathematical formulation that requires a time-dependent partitioning component rather than a reference FMI curve and does not call for an explicit allocation function. Without following indicated body composition, however, this formulation does not adjust the composition of weight changes to account for non-equilibrium initial conditions in body-composition (e.g. due to strength training) or deviations induced by weight gain/loss during childhood (e.g. the example above). While due to its extreme nature this simulation experiment may be infeasible to replicate empirically, the current model offers an alternative that may be more consistent with the general homeostatic mechanisms regulating human metabolism and growth. Case studies following body composition of obese children before and after significant weight loss, or malnourished children before and after significant weight gain, could settle this question empirically.

The last experiment compares the accuracy of body composition predictions between H2 and the current model. The H1 and H2 models are very close in simulating adulthood weight dynamics but only H2 is applicable to childhood and thus is used for this comparison. A random sample of 50 subjects with different ages from the NHANES data is taken. Fifty simulated individuals are created in each model, starting from ages 5 (for H2) and birth (for current) with a fixed fractional change (from reference) in energy intake that allows the individual to reach the observed weight-for-age in the corresponding NHANES sample. To keep the comparisons fair I do not calibrate the current model to match subjects' heights, instead, I use the average height for the current model in all cases. Figure 2-E graphs the empirically observed FFM for each subject against the predictions from H2 and the current model. The 45 degree line represents a perfect estimation of body composition. Both models do relatively well, with the current model outperforming H2 (Mean Absolute Percentage Error is 10% and 12.6% for the current and H2 models respectively; Root Mean Squared Error is 5.34 kg vs. 6.32 kg). This experiment also provides additional support for the use of the new partitioning formulation (Equations 1 and 2).

**Figure 3:**
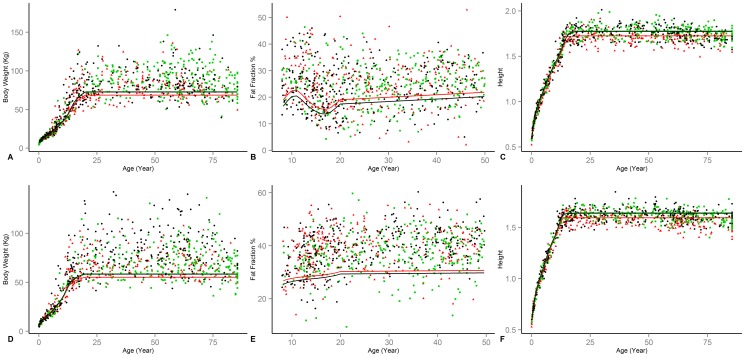
Comparison of base model and NHANES samples. Mexican American (Red triangles) and non-hispanic Black (Black Circle), and White (Green square). (A & D) Body weight for male and female; (B & E) Fat percentage for male and female; (C & F) Height for male and female.

Simulation results for height, weight, and body composition of a reference individual is shown in Figure 3, distinguished by gender (male on the first row, female on the second) and race (Mexican-American (Red triangles), Non-Hispanic white (Green square), and non-Hispanic black (Black circle)). Also graphed are random samples of 300 individuals from NHANES data for each race. While less obese than typical sampled individual, the simulated individuals' behavior over time is consistent with the empirical trends. The significant variability in the data also points to the importance of the model's potential for capturing the individual differences using both race and individual variation factors in height, FMI, BMI, and physical activity.

**Figure 4:**
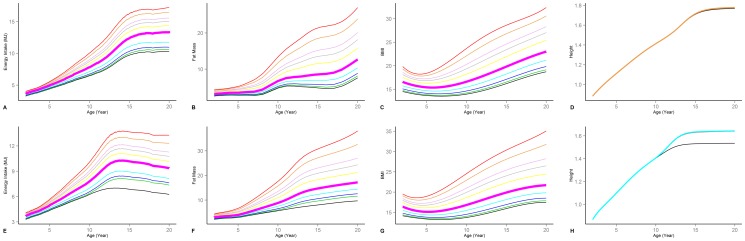
Growth trajectory for simulated subjects. Tracking 3^rd^, 5^th^, 10^th^, 25^th^, 50^th^, 75^th^, 85^th^, 90^th^, 95^th^, and 97^th^ CDC BMI percentiles. Median curves are bold and pink. Male and Female results for (A & E) Energy intake; (B & F) BMI; (C & G) Fat percentage; (D & H) Height (only 3^rd^, 5^th^, 10^th^, and 50^th^ percentiles are shown).

This model can be used for various applications. Two sets of analyses provide examples. First, the current model can be used to provide sample age, gender, and BMI specific reference body composition and energy intake curves. Whereas BMI percentile curves are readily available, reference curves for the corresponding body composition and energy intake for individuals not on the typical BMI reference are harder to obtain. The model can provide customized curves for individuals and groups. In Figure 4 EI, FM, BMI, and height trajectories (from left to right) are graphed for simulated individuals (none-hispanic white male (top row) and female (bottom row)) following different CDC BMI percentile curves [14]. EI for the 97^th^ BMI percentile individual grows to twice the 3^rd^ percentile female by age 20. Male variations are also significant, but more limited due to the smaller range in reference BMI curves. The corresponding FM curves show even more variation than BMI because the majority of weight difference is reflected in the changes in FM. Height curves show the impact of energy restriction on height, most notably for the 3^rd^ percentile female subject. Here, sustained reduction in energy intake required to keep the individual at the lower end of BMI distribution leads to stunted growth in height. While variations in EI over the years allows real subjects to benefit from catch up growth in periods of energy surplus, the simulated subject faces continuous energy deficit that significantly hurts her height growth.

**Figure 5:**
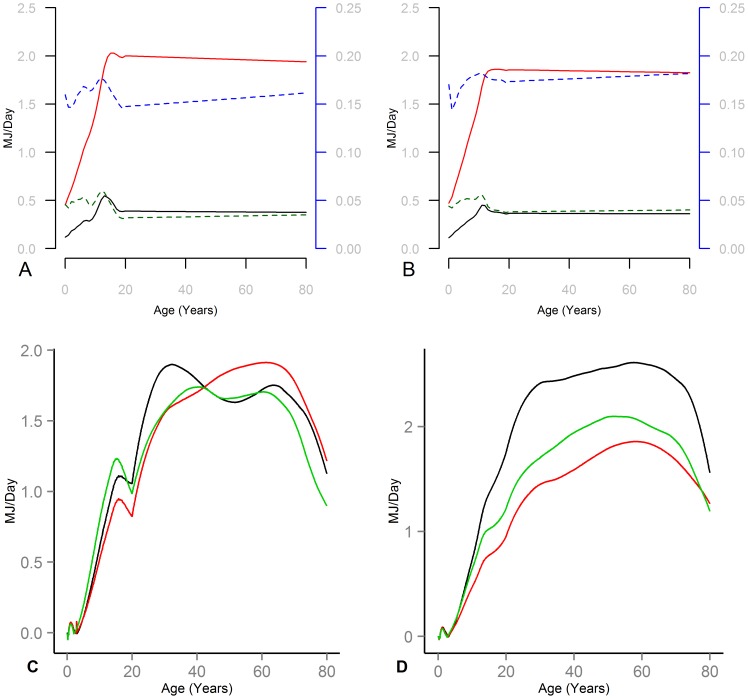
Energy gap analysis for different policy scenarios. (A & B) Male and Female; Absolute (left axis, solid lines) and fractional (dashed line, right axis) average daily energy deficit needed to reduce BMI by one (bottom curves) and five (top curves) units over a one year period for subjects at different ages. (C & D) Male and Female; Estimated energy surplus required by typical NHANES subject (Mexican American (green) and non-hispanic Black (black), and White (red)) compared to a subject following reference CDC BMIs.

Other applications include assessing energy gap needed for weight loss interventions and understanding different historical trends. Figure 5 reports on two example analyses. First, consider an individual one (or five) BMI unit(s) above reference curve and desiring to lose this extra weight over a one year period. What is the energy gap required to induce this weight change? Here the energy gap is defined as the average daily difference between the energy intake needed to keep the individual at initial BMI status (i.e. 1 or 5 units above reference for age) and EI that gets the individual to the reference BMI for age over exactly one year (calculated both in MJ/Day and as a fraction of daily EI). Using the model this energy gap is calculated for individuals at different ages and reported in absolute (solid lines, left axis) and fraction of EI (dashed, right axis) in panels A and B for male and female. Due to their higher height, adults require more energy gap to lose a unit of BMI than children. The fractional energy gap needed for similar BMI loss is more homogeneous across different ages, with variations due to differences in body composition across various ages and fraction of weight loss coming from FM. In a second experiment (Panels C and D) the maintenance energy gap, the energy surplus needed to maintain average individual's BMI at the level reflected in NHANES data, is compared to the energy needs if individuals followed CDC BMI reference curves. This is a measure of the magnitude of extra energy intake embedded in the population weight, in the absence of any further weight gain, and provides an estimate for the amount of change in the EI needed to take the population to healthy weights (Panels C and D for male and female). Average BMI curves are estimated for different gender and race groups using a kernel weighted local polynomial fit [42] across different ages to individual NHANES data. Consistent with the obesity epidemic the energy surpluses are positive across all groups and ages. Values are higher for middle aged adults and the drop among the elderly is consistent with lower obesity levels among very old as well as the reduction in BMR due to aging and changes in body composition. Moreover, two mechanisms reduce the childhood maintenance energy gap compared to adulthood. First, the gap between the reference and average BMI levels is significantly smaller in childhood. Average BMI difference across childhood ages is between 1.8 and 3.1 BMI units for children in various gender and ethnicity groups, compared to 5.2–9.7 BMI units for the adults. Moreover, extra mass in childhood reduces the growth energy needs, leading to lower maintenance energy gap. Male Mexican Americans (green lines) and female none-hispanic black (black lines) show the highest energy surplus in childhood vs. youth and adulthood, respectively. The magnitude of energy surplus, between 1–2 MJ/Day during adulthood, is large, and reaches 24% of total energy intake (averaged over all ages) for the female none-hispanic black. This measure is more than 14% among all other population groups as well, indicating the massive change needed in individual behaviors to return to a healthy population state.

## Discussion

The current model of human weight and height dynamics contributes to our understanding in a few different ways. First, this study introduces several novel extensions to the existing modeling literature in a single life-cycle model: it provides the first mathematical model of growth and body composition during infancy and early childhood; integrates the height dynamics with the weight and body composition modeling; and building on growth canalization and energy allocation offers an alternative architecture for modeling body weight, composition, and height with distinct features (e.g. return to indicated trajectories after any perturbation of body composition).

Mathematical models require a high degree of precision in identifying assumptions and their empirical basis. Thus, they allow us to integrate our knowledge and identify gaps. For example, in the development of the current model a few areas for further improvement are identified. First, the empirical evidence for formulating height change equations is thin. Longitudinal data from cases of malnutrition, stunting, and catch up growth can help bound the parameters of the model more accurately and suggest alternative specifications for the impact of energy availability on height growth rates. Second, the current empirical evidence does not fully specify adaptive thermogenesis. For example it is not clear if energy needs for catch up growth should be included in calculating the energy gap that drives adaptive thermogenesis (the current model includes those), or whether AT applies to the full energy gap or only the portion of it related to BMR (the assumption in the current model). Experiments that tease out these alternative mechanisms will be valuable. Third, in estimating RMR the change in the size of active body organs with the change in weight is estimated from cross sectional data, but would be more accurately estimated if future studies are conducted longitudinally, tracking organ size in the same individual as they gain or lose weight. Fourth, the existing body composition (and the resulting energy partitioning) equations are largely based on cross sectional data and samples between 8 and 50 years of age. Moreover, they do not account for physical activity, which, keeping everything else constant, may shift body composition. Longitudinal studies that track individuals' composition over time and across different ages (including infants and elderly), weights, and physical activity levels, can improve these equations.

A different use of models is in providing concrete and refutable predictions which can motivate empirical research [43]. For example, whereas the current model predicts a return to reference body composition curves after periods of weight gain and loss in children, H2 model suggests permanent changes in body composition are likely after such episodes. These predictions are based on different underlying mechanistic assumptions, e.g. whether there are homeostatic processes that bring body composition back to a reference, or the instantaneous partitioning of energy is the only mechanism behind the observed regularity in body composition. Such predictions can be tested using empirical case studies or experiments, and may identify otherwise unknown regulatory mechanisms.

The current model can also be used to provide projections for weight and height change in response to different nutrition and exercise programs [44]. From designing malnutrition treatment regiments in under-developed countries to assessing the effectiveness of different obesity interventions and policies in the U.S., such projections are integral to intervention design and the identification of the most cost-effective policies. Model outputs can be used to project population obesity trends expected from different energy gap scenarios or find energy gap responsible for an observed weight trajectory, among others. Projections can also inform clinical applications. For example the model can generate customized reference curves for growth across different sub-populations, assess energy needs for normal and catch up growth, and calibration to individual data can inform individualized diagnostics and intervention design. The current model provides such projections across all age groups and for any time horizon, though more detailed models [9], [10] may be more appropriate for assessing adult weight dynamics in shorter time horizons (hours to days).

The current model provides a modular structure that facilitates customization to different population groups and revision of the model based on new findings. Specifically, the CDC reference curves for BMI and Height may be replaced by reference curves from other populations providing a new customized model without a need to estimate other parameters. For example in collaboration with Healthy birth, growth and development knowledge integration researchers from the Bill & Melinda Gates Foundation, World Health Organization reference curves are being used in an ongoing study of child malnourishment. Reference FMI and Physical Activity curves could also be changed if there is reason to suspect significant differences in a new population. Finally, if future research finds body composition equations with better predictive power than those used here, the new equations could easily be plugged into the model.

The comparison of the current model against multiple empirical data sources as well as previous models provides confidence in the overall reliability of the results. Yet a few areas for further improvement are notable. First, the model can be expanded to better account for short-term dynamics that unfold over a few days. It may also be expanded to account for macro-nutrient composition of diet. These extensions are relatively simple and can build on the existing models that incorporate those features [9], [10]. Second, the current model is largely driven by exogenous reference curves for BMI, FMI, Height, and components of BMR. The underlying biological processes that generate these reference curves are inter-related and a more detailed modeling program can be defined to establish how, guided by the interactions of genes and environment, those curves result from the basic biological processes. Such extension is ambitious and requires a deeper understanding of diverse metabolic and growth mechanisms beyond our current knowledge, yet, it will provide a valuable synthesis and extension of current knowledge with potentially significant and diverse benefits. A more incremental extension includes estimating the joint distribution of individual-variation parameters for height, BMI, FMI, and PA. Such specification can help with representing a population of individuals more accurately.

Overall, besides integrating our current knowledge into a clearly specified mathematical representation that enables policy analysis, the current model highlights potential gaps in our knowledge that can guide future empirical studies and offers multiple avenues for policy and clinical application. I hope the fully documented model which follows simulation modeling reporting standards [45], with instructions for conducting independent analysis using the model, facilitate future research and practical applications of this research.

## Supporting Information

Appendix S1 **Model formulations.** Detailed model formulations with references to sources of data and assumptions.
(DOCX)

Appendix S2 **Comparison of different body composition equations.**
(DOCX)

Appendix S3 **Using the simulation model.** Instructions for using the simulation model in Appendix S4.
(DOCX)

Appendix S4 **Simulation model.** Vensim simulation models and supplementary files available for independent analysis and replication of results.
(ZIP)

## References

[1] Ogden CL, Carroll MD, Curtin LR, McDowell MA, Tabak CJ, et al (2006) Prevalence of overweight and obesity in the United States, 1999-2004. JAMA: The Journal Of The American Medical Association 295:1549–1555. 16595758 10.1001/jama.295.13.1549

[2] Wang Y, Lobstein T (2006) Worldwide trends in childhood overweight and obesity. Int J Pediatr Obes 1:11–25. 17902211 10.1080/17477160600586747

[3] Swinburn BA, Sacks G, Hall KD, McPherson K, Finegood DT, et al (2011) The global obesity pandemic: shaped by global drivers and local environments. Lancet 378:804–814. 21872749 10.1016/S0140-6736(11)60813-1

[4] Wang YC, McPherson K, Marsh T, Gortmaker SL, Brown M (2011) Health and economic burden of the projected obesity trends in the USA and the UK. Lancet 378:815–825. 21872750 10.1016/S0140-6736(11)60814-3

[5] Hall KD (2008) What is the required energy deficit per unit weight loss? International Journal of Obesity 32:573–576. 17848938 10.1038/sj.ijo.0803720 PMC2376744

[6] Christiansen E, Garby L, Sørensen TIA (2005) Quantitative analysis of the energy requirements for development of obesity. Journal of Theoretical Biology 234:99–106. 15721039 10.1016/j.jtbi.2004.11.012

[7] Flatt J-P (2004) Carbohydrate-Fat Interactions and Obesity Examined by a Two-Compartment Computer Model. Obesity 12:2013–2022. 10.1038/oby.2004.252 15687403

[8] Hall KD, Sacks G, Chandramohan D, Chow CC, Wang YC, et al (2011) Obesity 3 Quantification of the effect of energy imbalance on bodyweight. Lancet 378:826–837. 21872751 10.1016/S0140-6736(11)60812-X PMC3880593

[9] Hall KD (2010) Predicting metabolic adaptation, body weight change, and energy intake in humans. American Journal Of Physiology Endocrinology And Metabolism 298:E449–E466. 19934407 10.1152/ajpendo.00559.2009 PMC2838532

[10] Hall KD, Sacks G, Chandramohan D, Chow CC, Wang YC, et al (2011) Quantification of the effect of energy imbalance on bodyweight. Lancet 378:826–837. 21872751 10.1016/S0140-6736(11)60812-X PMC3880593

[11] Butte NF, Christiansen E, Sorensen TIA (2007) Energy imbalance underlying the development of childhood obesity. Obesity 15:3056–3066. 18198315 10.1038/oby.2007.364

[12] Hall KD, Butte NF, Swinburn BA, Chow CC (2013) Quantifying the Dynamics of Childhood Growth and Obesity. Lancet Diabetes and Endocrinology 1:97–105. 24349967 10.1016/s2213-8587(13)70051-2 PMC3857695

[13] Waddington CH (1957) The strategy of the genes; a discussion of some aspects of theoretical biology. London,: Allen & Unwin. ix262 pp.

[14] Kuczmarski RJ, Ogden CL, Grummer-Strawn LM, Flegal KM, Guo SS, et al. (2000) CDC growth charts: United States. Adv Data: 1–27. 11183293

[15] CDC (2013) National Health and Nutrition Examination Survey. Centers for Disease Control and Prevention.

[16] Wells JC, Williams JE, Chomtho S, Darch T, Grijalva-Eternod C, et al (2012) Body-composition reference data for simple and reference techniques and a 4-component model: a new UK reference child. Am J Clin Nutr 96:1316–1326. 23076617 10.3945/ajcn.112.036970

[17] Butte NF, Hopkinson JM, Wong WW, Smith EO, Ellis KJ (2000) Body composition during the first 2 years of life: an updated reference. Pediatr Res 47:578–585. 10813580 10.1203/00006450-200005000-00004

[18] Forbes GB (1987) Lean body mass-body fat interrelationships in humans. Nutr Rev 45:225–231. 3306482 10.1111/j.1753-4887.1987.tb02684.x

[19] Altman PL, Dittmer DS (1962) Growth including reproduction and morphological development. Washington,: Federation of American Societies for Experimental Biology. xii, 608 p.

[20] Wang ZM (2012) High ratio of resting energy expenditure to body mass in childhood and adolescence: A mechanistic model. American Journal of Human Biology 24:460–467. 22368150 10.1002/ajhb.22246

[21] Torun B (2005) Energy requirements of children and adolescents. Public Health Nutr 8:968–993. 16277815 10.1079/phn2005791

[22] Doucet E, St-Pierre S, Almeras N, Despres JP, Bouchard C, et al (2001) Evidence for the existence of adaptive thermogenesis during weight loss. Br J Nutr 85:715–723. 11430776 10.1079/bjn2001348

[23] Wit JM, Boersma B (2002) Catch-up growth: definition, mechanisms, and models. J Pediatr Endocrinol Metab 15 Suppl 5: 1229–1241. 12510974

[24] Boersma B, Wit JM (1997) Catch-up growth. Endocr Rev 18:646–661. 9331546 10.1210/edrv.18.5.0313

[25] Walker SP, Golden MH (1988) Growth in length of children recovering from severe malnutrition. Eur J Clin Nutr 42:395–404. 3135181

[26] Tennefors C, Coward WA, Hernell O, Wright A, Forsum E (2003) Total energy expenditure and physical activity level in healthy young Swedish children 9 or 14 months of age. European Journal of Clinical Nutrition 57:647–653. 12771965 10.1038/sj.ejcn.1601591

[27] Visser M, Deurenberg P, Vanstaveren WA, Hautvast JGAJ (1995) Resting Metabolic-Rate and Diet-Induced Thermogenesis in Young and Elderly Subjects - Relationship with Body-Composition, Fat Distribution, and Physical-Activity Level. American Journal of Clinical Nutrition 61:772–778. 7702018 10.1093/ajcn/61.4.772

[28] Salbe AD, Weyer C, Harper I, Lindsay RS, Ravussin E, et al (2002) Assessing risk factors for obesity between childhood and adolescence: II. Energy metabolism and physical activity. Pediatrics 110:307–314. 12165583 10.1542/peds.110.2.307

[29] Spadano JL, Bandini LG, Must A, Dallal GE, Dietz WH (2005) Longitudinal changes in energy expenditure in girls from late childhood through midadolescence. American Journal of Clinical Nutrition 81:1102–1109. 15883435 10.1093/ajcn/81.5.1102

[30] Wang Z, Heshka S, Heymsfield SB, Shen W, Gallagher D (2005) A cellular-level approach to predicting resting energy expenditure across the adult years. Am J Clin Nutr 81:799–806. 15817855 10.1093/ajcn/81.4.799

[31] Luhrmann PM, Herbert BM, Neuhauser-Berthold M (2001) Effects of fat mass and body fat distribution on resting metabolic rate in the elderly. Metabolism-Clinical and Experimental 50:972–975. 11474487 10.1053/meta.2001.24871

[32] Wells JCK, Fewtrell MS, Williams JE, Haroun D, Lawson MS, et al (2006) Body composition in normal weight, overweight and obese children: matched case-control analyses of total and regional tissue masses, and body composition trends in relation to relative weight. International Journal of Obesity 30:1506–1513. 16770333 10.1038/sj.ijo.0803402

[33] Fields DA, Krishnan S, Wisniewski AB (2009) Sex Differences in Body Composition Early in Life. Gender Medicine 6:369–375. 19682664 10.1016/j.genm.2009.07.003

[34] Carberry AE, Colditz PB, Lingwood BE (2010) Body Composition From Birth to 4.5 Months in Infants Born to Non-Obese Women. Pediatric Research 68:84–88. 20351656 10.1203/PDR.0b013e3181df5421

[35] Butte NF (2005) Energy requirements of infants. Public Health Nutrition 8:953–967. 16277814 10.1079/phn2005790

[36] Torun B (2005) Energy requirements of children and adolescents. Public Health Nutrition 8:968–993. 16277815 10.1079/phn2005791

[37] Ellis KJ, Shypailo RJ, Abrams SA, Wong WW (2000) The reference child and adolescent models of body composition - A contemporary comparison. In Vivo Body Composition Studies 904:374–382. 10.1111/j.1749-6632.2000.tb06486.x 10865775

[38] Talbot FB (1938) Basal metabolism standards for children. American Journal of Diseases of Children 55:455–459.

[39] Poehlman ET, Berke EM, Joseph JR, Gardner AW, Katzmanrooks SM, et al (1992) Influence of Aerobic Capacity, Body-Composition, and Thyroid-Hormones on the Age-Related Decline in Resting Metabolic-Rate. Metabolism-Clinical and Experimental 41:915–921. 1640872 10.1016/0026-0495(92)90177-c

[40] Poehlman ET, Goran MI, Gardner AW, Ades PA, Arciero PJ, et al (1993) Determinants of Decline in Resting Metabolic-Rate in Aging Females. American Journal of Physiology 264:E450–E455. 8460693 10.1152/ajpendo.1993.264.3.E450

[41] Hall KD (2007) Body fat and fat-free mass inter-relationships: Forbes's theory revisited. Br J Nutr 97:1059–1063. 17367567 10.1017/S0007114507691946 PMC2376748

[42] Gutierrez RG, Linhart JM, Pitblado JS (2003) From the help desk: Local polynomial regression and Stata plugins. Stata Journal 412–419.

[43] Gobel B, Sanghvi A, Hall KD (2014) Quantifying energy intake changes during obesity pharmacotherapy. Obesity 22:2105–2108. 24961931 10.1002/oby.20813 PMC4180778

[44] Brady I, Hall KD (2014) Dispatch from the Field: Is Mathematical Modeling Applicable to Obesity Treatment in the Real World? Obesity 22:1939–1941. 24895253 10.1002/oby.20804 PMC4149602

[45] Rahmandad H, Sterman J (2012) Reporting Guidelines for Simulation-based Research in Social Sciences International System Dynamics Conference. Saint Gallan, Switzerland.

