# Supplementary material for: Human Growth and Body Weight Dynamics: An Integrative Systems Model
Source: PLoS One. 2014 Dec 5;9(12):e114609. doi: 10.1371/journal.pone.0114609 (PMC4257729; doi:10.1371/journal.pone.0114609)
Supplement: Appendix S1 — Model formulations. Detailed model formulations with references to sources of data and assumptions. (DOCX) [file pone.0114609.s001.docx]

### A- Model formulations

FM, FFM and H are the three state variables that identify the current model. In the following I document the model by explaining how the rates of change in each are determined. For simplicity I exclude the equations needed to generate variation across individuals. However those are available in the full model equations which are documented in the Vensim ™ model and provided along with this submission. Additional information for opening these model files using free Vensim model reader and conducting simulation analysis on them is also provided to enable independent inspection and analysis (See Appendix C).

At the heart of the model is the matching of energy supply and demand. I divide the sources of demand and supply into five items each. This distinction is helpful in assigning the priorities needed for the allocation of energy supply to demanding processes. The energy supply sources and their equations are summarized in the table below:

Table 1- Potential sources of energy and their equations.

| **Source** | **Equation** | **Eq #** |
| --- | --- | --- |
| Energy Intake | $S_{EI}=EI$ | S1 |
| Metabolizing extra fat mass | $S_{ExtF}=Max\left( 0,-\left( \rho_{F}+\eta_{F} \right).\left( \frac{dFM}{dt} \right)^{*} \right)+Max\left( 0,-\left( \rho_{F}+\eta_{F} \right).\left( \frac{dFM}{dt} \right)_{Bal} \right)$ | S2 |
| Metabolizing extra fat free mass | $S_{ExtL}=Max\left( 0,-\left( \rho_{L}+\eta_{L} \right).\left( \frac{dFFM}{dt} \right)^{*} \right)+Max\left( 0,-\left( \rho_{L}+\eta_{L} \right).\left( \frac{dFFM}{dt} \right)_{Bal} \right)$ | S3 |
| Metabolizing essential fat mass | $S_{EssF}=\left( \rho_{F}+\eta_{F} \right).\frac{FM}{\tau}.\left( 1-C \right)-S_{ExtF}$ | S4 |
| Metabolizing essential fat free mass | $S_{EssL}=\left( \rho_{L}+\eta_{L} \right).\frac{FFM}{\tau}.C-S_{ExtL}$ | S5 |

Two distinct sources of energy can contribute to supply of energy from “extra” mass. The indicated mass velocities for growth $\left( \frac{\mathrm{dFM}}{\mathrm{dt}} \right)^{*}$and $\left( \frac{\mathrm{dFFM}}{\mathrm{dt}} \right)^{*}$are based on indicated growth trajectories ( based on FM* and FFM*), and if negative, supply extra energy, indicating an over-weight person whose body mass beyond indicated levels would provide surplus energy. A second component of extra mass energy supply is due to the balancing mass velocities $\left( \frac{\mathrm{dFM}}{\mathrm{dt}} \right)_{\mathrm{Bal}}$and $\left( \frac{\mathrm{dFFM}}{\mathrm{dt}} \right)_{\mathrm{Bal}}$. These quantities are determined by comparing actual FM and the indicated FM for the current BMI and Height. A mechanism that attempts to correct imbalances in body composition over τ will create these two additional sources. Equations for these four factors are discussed further down in the text. Together, these provide energy supply from metabolizing the extra mass, while consuming the remaining (essential) FM and FFM provides a last resort when EI falls short of supporting maintenance energy needs (Equations S2-S5). Parameters $\rho$ and $\eta$ specify the energy density and energy costs of synthesis of one Kg of mass and are specified based on the literature (see Table 5). Note that $\rho_{L}$ is a linear function of FFM ([1](#_ENREF_1)):

$\rho_{L}=Min({\rho_{L}}^{Adult}, {\rho_{L}}^{Base}+\alpha_{\rho}.FFM)$ S6

The energy partitioning factor C specifies what fraction of the energy is to be supplied from essential body mass comes from FFM and can be represented as in equation S7. Factor $\left( \frac{\mathrm{dFFM}}{\mathrm{dBW}} \right)$ is specified based on the reference FMI for the BMI equation and discussed further down in the text:.

$C=1-\frac{1}{\frac{\left( \rho_{L}+\eta_{L} \right)\left( \frac{dFFM}{dBW} \right)}{\left( \rho_{F}+\eta_{F} \right)\left( 1-\frac{dFFM}{dBW} \right)}+1}$ S7

Energy demand is similarly categorized into five items:

Table 2- Potential demands for energy and their equations.

| **Energy demand for:** | **Equation** | **Eq #** |
| --- | --- | --- |
| Maintenance | $D_{M}={BMR}_{D}+PA.BW+\beta.EI+\beta_{T}.\Delta EI$ | S8 |
| Creating essential fat mass | $D_{EssF}=Max(0,(\rho_{F}+\eta_{F}).\left( \frac{dFM}{dt} \right)^{*}) +Max\left( 0,\left( \rho_{F}+\eta_{F} \right).\left( \frac{dFM}{dt} \right)_{Bal} \right)$ | S9 |
| Creating essential fat free mass | $D_{EssL}=Max(0,(\rho_{L}+\eta_{L}).\left( \frac{dFFM}{dt} \right)^{*}) +Max\left( 0,\left( \rho_{L}+\eta_{L} \right).\left( \frac{dFFM}{dt} \right)_{Bal} \right)$ | S10 |
| Creating extra fat mass | $D_{ExtF}=Max(0,\left( 1-C \right)\left( S_{EI}+S_{ExtF}+S_{ExtL}-D_{M}-D_{EssF}-D_{EssL} \right))$ | S11 |
| Creating extra fat free mass | $D_{ExtL}=Max(0,C.\left( S_{EI}+S_{ExtF}+S_{ExtL}-D_{M}-D_{EssF}-D_{EssL} \right))$ | S12 |

The maintenance component of energy demand includes contributions of basal metabolic rate, physical activity, thermic effect of feeding, and adaptive thermogenesis. Throughout the analysis reference physical activity values: ${PA}^{Ref}=(({PAL}^{Ref}(1-\beta)-1)*{BMR}_{Ref})/{BW}_{Ref}$ S13

are used unless specified. Note that PA values use the BW and BMR for a reference individual (BW_Ref_ and BMR_Ref_) rather than the current BW/BMR for the individual, because the latter formulation will introduce a feedback between weight change dynamics and physical activity: gaining weight will decrease PA values automatically, which confounds different mechanisms. In fact use of PAL values is not generally recommended given the complications discussed and is only employed due to its common use in the literature.

The adaptive thermogenesis demand is based on the ratio of actual vs. equilibrium energy intake when applied to the BMR component of energy expenditure:

$\Delta EI=\frac{EI}{{EI}^{eq}}{BMR}_{D}$ S14

The BMR component of demand adjusts base BMR for an individual based on the relative cellularity for the age (${CL}^{Ref}$) and required adjustments for variations of FFM and FM from reference values:

${BMR}_{D}={CL}^{Ref}({BMR}^{Base}+\gamma_{\Delta L}.{FFM}^{Adj}+\gamma_{F}.{FM}^{Adj})$ S15

Base BMR calculates the BMR contribution of Brain, Kidneys, Liver, Heart, and other tissues based on organ specific weights (${M_{i}}^{Ref}$; i=1..5 respectively) and their corresponding daily energy needs per Kg ($\gamma_{i}$), scaled to the individuals reference weight for height. A single curve for the fractional weight of four active organs (sum in equation S17) is estimated based on data from Altman and Dittmer ([4](#_ENREF_4)) and a parametric curve for energy requirements of the four organs per unit of weight (sum in equation S17) is estimated based on data in Wang’s study ([5](#_ENREF_5)). ${FFM}^{Adj}$ and ${FM}^{Adj}$ are deviations from reference values for FM and FFM, after taking into account the fat mass fraction of active metabolic organs ($f_{FM}$). The energy need of FFM deviations is determined based on mass change in each type of organ with one unit of change in body weight, and their corresponding energy needs ([6](#_ENREF_6)). FM deviations exclude the fat mass in active organs to avoid double counting:

${BMR}^{Base}=\left( \sum_{i=1}^{5} \gamma_{i}\frac{{M_{i}}^{Ref}}{{BW}^{Ref}} \right).H^{2}.{BMI}^{Ref}$ S16

${FM}^{Adj}=FM-f_{FM}.\left( \sum_{i=1}^{4} \frac{{M_{i}}^{Ref}}{{BW}^{Ref}} \right).H^{2}.{BMI}^{Ref}$ S17

${FFM}^{Adj}=FFM-H^{2}.({BMI}^{Ref}-{FMI}^{Ref})$ S18

$\gamma_{\Delta L}=\sum_{i=1}^{5} \gamma_{i}\frac{dM_{i}}{dBW}$ S19

Once the supply and demand for energy are determined, I use a priority-based allocation algorithm to specify which supply sources become active and how much each demand item is satisfied. Mathematical details for this allocation function are documented elsewhere^[[1]](#footnote-1)^. The basic idea is that different demand/supply sources have different priorities which specify which source of energy supply can service which demanding process in the body. The resulting demand/supply source matching is reported in Table 3. In each cell of the table I identify if the given supply source (row) may supply energy to the given demand source (column). For example body will use supply from EI ($S_{EI}$) to satisfy maintenance energy demand ($D_{M}$) with no reservation, thus the Y marker in the relevant cell. N means the priority of supply source is higher than demand, and therefore no supply will be provided from this supply source to the corresponding demand (e.g. essential FFM will not be used to generate reserve FM). The output of this function includes the amount of each demanding component that is satisfied, $D^{B}$(subscripted for the five sources of demand), and contribution of each supplying source, $S^{B}$(subscripted for the five sources of supply), where superscript *B* indicates the energy balancing values from the allocation process.

Table 3- Priorities of different energy supply and demand sources in energy allocation process.

| **Demand**  **Supply** | $\boldsymbol{D}_{\boldsymbol{M}}$ | $\boldsymbol{D}_{\boldsymbol{EssL}}$ | $\boldsymbol{D}_{\boldsymbol{EssF}}$ | $\boldsymbol{D}_{\boldsymbol{ExtL}}$ | $\boldsymbol{D}_{\boldsymbol{ExtF}}$ |
| --- | --- | --- | --- | --- | --- |
| $S_{EI}$ | Y | Y | Y | Y | Y |
| $S_{EssL}$ | Y | N | N | NA | N |
| $S_{EssF}$ | Y | N | N | N | NA |
| $S_{ExtL}$ | Y | NA | Y | NA | N |
| $S_{ExtF}$ | Y | Y | NA | N | NA |

Indicated FM and FFM velocity, $\left( \frac{dFM}{dt} \right)^{*}$ and $\left( \frac{dFFM}{dt} \right)^{*}$, are determined based on comparison of current FM and FFM with their indicated values d time units ahead. The model is insensitive to look-forward horizon, d, as long as it is small enough. Those indicated values are a function of indicated FMI (FMI*) and H*. FMI* is specified based on equations estimated by regressions on NHANES data, detailed in equations S22 and S23. Reference FMI (${FMI}^{Ref}$) is coming from two different studies for infant and children ([7](#_ENREF_7), [8](#_ENREF_8)) with interpolation between the age spans covered by two using slopes found in ([9](#_ENREF_9)); the reference values then linearly increase with age during adulthood based on trends in NHANES data and these values are summarized in Table 4.

$\left( \frac{dFM}{dt} \right)^{*}(t)=\frac{{(FMI}^{*}(t+d).{H^{*}\left( t+d \right)}^{2}-FM(t))}{d}$ S20

$\left( \frac{dFFM}{dt} \right)^{*}(t)=\frac{({BMI}^{*}(t+d)-{FMI}^{*}(t+d)).{H^{*}\left( t+d \right)}^{2}-FFM(t))}{d}$ S21

${{FMI}_{Fem}}^{*}={{FMI}_{Fem}}^{Ref}+0.54.({BMI}^{*}-{{BMI}_{Fem}}^{Ref})+0.0029.{({BMI}^{*}-{{BMI}_{Fem}}^{Ref})}^{2}+0.20.MxAm$ S22

${{FMI}_{Male}}^{*}={{FMI}_{Male}}^{Ref}+0.40.({BMI}^{*}-{{BMI}_{Male}}^{Ref})+0.0097.{({BMI}^{*}-{{BMI}_{Male}}^{Ref})}^{2}+0.37.MxAm$ S23

Here MxAm is one if the individual is Mexican American, and 0 otherwise.

The balancing FM and FFM velocities, $\left( \frac{\mathrm{dFM}}{\mathrm{dt}} \right)_{\mathrm{Bal}}$ and $\left( \frac{\mathrm{dFFM}}{\mathrm{dt}} \right)_{\mathrm{Bal}}$ are specified by comparing current FM and FFM against the indicated values for the current BMI (FMI_Bal_), using a similar equation as S22 and S23, but applied with the current, rather than indicated, BMI. Any potential gap is then corrected over a time horizon of τ and given than this component only adjusts body composition for the current BMI, the balancing velocities for FFM is the negative of the same concept for FM.

$\left( \frac{\mathrm{dFM}}{\mathrm{dt}} \right)_{\mathrm{Bal}}=\frac{\left( {FMI}_{Bal}-FMI \right)H^{2}}{\tau}$ S24

$\left( \frac{\mathrm{dFFM}}{\mathrm{dt}} \right)_{\mathrm{Bal}}=-\left( \frac{\mathrm{dFM}}{\mathrm{dt}} \right)_{\mathrm{Bal}}$ S25

${FMI}_{Fem\_Bal}={{FMI}_{Fem}}^{Ref}+0.54.(BMI-{{BMI}_{Fem}}^{Ref})+0.0029.{(BMI-{{BMI}_{Fem}}^{Ref})}^{2}+0.20.MxAm$ S26

${FMI}_{Male\_Bal}={{FMI}_{Male}}^{Ref}+0.40.(BMI-{{BMI}_{Male}}^{Ref})+0.0097.{(BMI-{{BMI}_{Male}}^{Ref})}^{2}+0.37.MxAm$ S27

The partitioning factor $\left( \frac{dFFM}{dBW} \right)$ is obtained by taking derivatives from equations S22 and S23, and solving for the speed of change in FM as a result of a change in BW:

$\left( \frac{dFFM}{dBW} \right)_{Fem}=1-Min(1,0.54+0.0058(BMI-{{BMI}_{Fem}}^{Ref}))$ S28

$\left( \frac{dFFM}{dBW} \right)_{Male}=1-Min(1,0.4+0.0194\left( BMI-{{BMI}_{Fem}}^{Ref} \right))$ S29

Indicated BMI, ${BMI}^{*}$, is assumed a weighted average of current BMI and the reference value, ${BMI}^{Ref}$, taken from CDC growth charts for ages under 20, and staying at the value for 20-year-olds for the rest of the life. The weighting parameter, sb, indicates the extent of natural pressure to adjust energy demand (and as a result energy intake, in equilibrium conditions) to shift the weight towards a reference, normal, level. As long as *sb* is none-zero, the indicated BMI moves towards that reference values and the dynamics of the model are not very sensitive to this parameter. A value of zero will remove the impact of reference BMI from the model and allows the BMI (and thus weight) move with no anchor in any biologically preferred normal range. In the reported simulations we use sb=0.5, which allows current BMI to be a major determinant of indicated value, but keeps the corrective pressure of reference values.

${BMI}^{*}\left( t+d \right)={BMI}^{Ref}\left( t+d \right).sb+BMI\left( t \right).\left( 1-sb \right)$ S30

Finally, FM and FFM change according to the allocated energy from equations above that balances the total supply and demand for energy. Specifically:

$\frac{dFFM}{dt}=\frac{\left( {D_{ExtL}}^{B}+{D_{EssL}}^{B}-{S_{ExtL}}^{B}-{S_{EssL}}^{B} \right)}{(\rho_{L}+\eta_{L})}$ S31

$\frac{dFM}{dt}=\frac{\left( {D_{ExtF}}^{B}+{D_{EssF}}^{B}-{S_{ExtF}}^{B}-{S_{EssF}}^{B} \right)}{(\rho_{F}+\eta_{F})}$ S32

The remaining equations specify the dynamics of height and how that depends on weight dynamics.

$\frac{dH}{dt}=Min\left( \left( \frac{dH}{dt} \right)^{*},\left( \frac{dH}{dt} \right)^{Max} \right).Max(0,Min(1,\left( \frac{BMI}{{BMI}^{Ref}}-0.85 \right).s_{H}+0.5))$ S33

Here the term $\left( \frac{dH}{dt} \right)^{*}(t)$ represents the indicated height velocity at age *a*, based on the equation S28, where $H^{*}$ values are driven from reference values of CDC growth charts, $H^{Ref}$:

$\left( \frac{dH}{dt} \right)^{*}(t)=\frac{H^{*}\left( t+d \right)-H(t)}{d}$ S34

The next term, $\left( \frac{dH}{dt} \right)^{Max}$, specifies the maximum catch-up growth rate feasible, which is approximated to be $v_{H}=4$ times the normal height velocity for the age ([10](#_ENREF_10)):

$\left( \frac{dH}{dt} \right)^{Max}(t)=v_{H}.\frac{H^{*}\left( t+d \right)-H^{*}(t)}{d}$ S35

The last term in rate of change in height specifies how the body allocates energy to catch up growth in height vs. essential body mass. The basic idea is that catch up growth will only start when a minimum level of BMI is achieved. A linear relationship with slope $s_{H}$ and centered around a BMI to reference BMI ratio of 0.85 ([11](#_ENREF_11)) is assumed to regulate this effect. Empirical estimates for parameter $s_{H}$ are not available; a moderate value of 8 is chosen and the impact on reported results are minimal.

Overall, three exogenous reference curves, $H^{Ref},{BMI}^{Ref}$and ${FMI}^{Ref}$ are used to specify the canalization of the growth for each gender (these curves are specified in Table 4). Reference cellularity and organ sizes are used to drive BMR values mechanistically and without leaving any free parameters in energy expenditure for fitting the individual data. This set up provides a strong test in assessing model’s fit to BMR and Total Energy Expenditure data, while allowing for customization of the model to specific individuals through variations in H*, FM* and FFM* (not discussed in this paper). The dynamic variations around these curves are controlled through the equations discussed above; the key equations to understand those variations are S22 and S23, which reflect the indicated partitioning of body mass into FM and FFM, and the processes for growth stunting and catch up (S27-S29). The model follows normal growth trajectory (and equilibrium in adulthood) by setting:

${EI}^{eq}={BMR}_{D}+PA.BW+\beta.EI+D_{EssF}+D_{EssL}$ S36

Consistent with most previous models this definition of equilibrium keeps an overweight adult at the same BMI level. Yet an underweight person will gain weight until she reaches the reference weight, i.e. more consistent with a growth-based view of equilibrium, due to inclusion of terms $D_{EssF}+D_{EssL}$. Depending on the purpose, these terms could be removed for adults, letting underweight individuals to stay at their BMI, or energy supply from extra mass ($S_{ExtF}+S_{ExtL}$) could be deducted from the above equation to allow overweight people to also move towards reference values.

The reference input series and the parameters that complete the definition of the model are listed in Table 4 and Table 5. Linear interpolation is used for the reference curves between two data points. Note that all the reference curves and most of the parameters are taken from the literature and no parameter is calibrated to match any specific data series. The impact of one free parameter, *sb*, is rather small and largely in changing the endogenous EI values off the reference trajectory. I found no data to estimate the other, $s_{H}$, so it is set so that low BMI ceases to slow height velocity when $\frac{BMI}{{BMI}^{Ref}}$ reaches 91%. The main impact of this parameter is only seen under simulations of significant and continued malnourishment, which is not the focus of the current paper. Nevertheless further studies for estimation of this parameter are important.

Table 4- Reference input values used in simulating the model.

| **Reference Variable** | **Reference values** | **Source** |
| --- | --- | --- |
| ${{BMI}_{Fem}}^{Ref}$ | (Age in Year, Reference BMI in Kg/M^2^) (0.0417,14.2),(0.125,14.9),(0.208,15.5),(0.292,16),(0.375,16.5),(0.458,16.8),(0.542,17.1),(0.625,17.2),(0.708,17.4),(0.792,17.4),(0.875,17.5),(0.958,17.5),(1.04,17.5),(1.13,17.4),(1.21,17.4),(1.29,17.3),(1.38,17.2),(1.46,17.1),(1.54,17),(1.63,16.9),(1.71,16.8),(1.79,16.7),(1.88,16.5),(1.96,16.4),(2,16.4),(2.29,16.2),(2.79,15.8),(3.29,15.6),(3.79,15.4),(4.29,15.2),(4.79,15.2),(5.29,15.2),(5.79,15.2),(6.29,15.3),(6.79,15.4),(7.29,15.5),(7.79,15.7),(8.29,15.9),(8.79,16.2),(9.29,16.4),(9.79,16.7),(10.3,17),(10.8,17.3),(11.3,17.6),(11.8,17.9),(12.3,18.3),(12.8,18.6),(13.3,18.9),(13.8,19.2),(14.3,19.5),(14.8,19.8),(15.3,20.1),(15.8,20.3),(16.3,20.6),(16.8,20.8),(17.3,21),(17.8,21.2),(18.3,21.4),(18.8,21.5),(19.3,21.6),(19.8,21.7),(20,21.7) | ([12](#_ENREF_12)) |
| ${{BMI}_{Mal}}^{Ref}$ | (Age in Year, Reference BMI in Kg/M^2^) (0.0417,14.4),(0.125,15.2),(0.208,16),(0.292,16.6),(0.375,17.1),(0.458,17.5),(0.542,17.7),(0.625,17.9),(0.708,18),(0.792,18.1),(0.875,18.1),(0.958,18.1),(1.04,18.1),(1.13,18),(1.21,17.9),(1.29,17.8),(1.38,17.6),(1.46,17.5),(1.54,17.4),(1.63,17.2),(1.71,17.1),(1.79,17),(1.88,16.8),(1.96,16.7),(2,16.6),(2.29,16.4),(2.79,16.1),(3.29,15.9),(3.79,15.7),(4.29,15.6),(4.79,15.5),(5.29,15.4),(5.79,15.4),(6.29,15.4),(6.79,15.5),(7.29,15.6),(7.79,15.7),(8.29,15.9),(8.79,16.1),(9.29,16.3),(9.79,16.5),(10.3,16.8),(10.8,17.1),(11.3,17.3),(11.8,17.7),(12.3,18),(12.8,18.3),(13.3,18.6),(13.8,19),(14.3,19.3),(14.8,19.7),(15.3,20),(15.8,20.4),(16.3,20.7),(16.8,21.1),(17.3,21.4),(17.8,21.7),(18.3,22.1),(18.8,22.4),(19.3,22.6),(19.8,22.9),(20,23) |  |
| ${H_{Fem}}^{Ref}$ | (Age in Year, Reference Height in cm) (0.0417,51.7),(0.125,55.3),(0.208,58.1),(0.292,60.5),(0.375,62.5),(0.458,64.4),(0.542,66.1),(0.625,67.7),(0.708,69.2),(0.792,70.6),(0.875,71.9),(0.958,73.2),(1.04,74.4),(1.13,75.6),(1.21,76.7),(1.29,77.8),(1.38,78.8),(1.46,79.8),(1.54,80.8),(1.63,81.8),(1.71,82.7),(1.79,83.6),(1.88,84.5),(1.96,85.3),(2,85),(2.29,88),(2.79,92.4),(3.29,95.9),(3.79,99.3),(4.29,103),(4.79,106),(5.29,110),(5.79,113),(6.29,117),(6.79,120),(7.29,123),(7.79,126),(8.29,129),(8.79,132),(9.29,134),(9.79,137),(10.3,140),(10.8,143),(11.3,146),(11.8,150),(12.3,153),(12.8,156),(13.3,158),(13.8,160),(14.3,161),(14.8,162),(15.3,162),(15.8,162),(16.3,163),(16.8,163),(17.3,163),(17.8,163),(18.3,163),(18.8,163),(19.3,163),(19.8,163) | ([12](#_ENREF_12)) |
| ${H_{Mal}}^{Ref}$ | (Age in Year, Reference Height in cm) (0.0417,52.7),(0.125,56.6),(0.208,59.6),(0.292,62.1),(0.375,64.2),(0.458,66.1),(0.542,67.9),(0.625,69.5),(0.708,70.9),(0.792,72.3),(0.875,73.7),(0.958,74.9),(1.04,76.1),(1.13,77.3),(1.21,78.4),(1.29,79.4),(1.38,80.5),(1.46,81.4),(1.54,82.4),(1.63,83.3),(1.71,84.2),(1.79,85.1),(1.88,86),(1.96,86.8),(2,86.5),(2.29,89.2),(2.79,93.4),(3.29,97.2),(3.79,101),(4.29,104),(4.79,108),(5.29,111),(5.79,114),(6.29,117),(6.79,120),(7.29,124),(7.79,127),(8.29,130),(8.79,132),(9.29,135),(9.79,138),(10.3,140),(10.8,142),(11.3,145),(11.8,148),(12.3,151),(12.8,154),(13.3,158),(13.8,162),(14.3,166),(14.8,169),(15.3,171),(15.8,173),(16.3,174),(16.8,175),(17.3,176),(17.8,176),(18.3,176),(18.8,177),(19.3,177),(19.8,177) |  |
| ${{FMI}_{Fem}}^{Ref}$ | (Age in Year, Reference FMI in Kg/M^2^) (0,1.92),(0.25,5.16),(0.5,5.52),(0.75,4.9),(1,4.62),(1.5,4.27),(2,3.97),(3,3.66),(4,3.38),(5,3.3),(6,3.54),(7,3.77),(8,4.01),(9,4.24),(10,4.46),(11,4.68),(12,4.9),(13,5.11),(14,5.32),(15,5.51),(16,5.7),(17,5.88),(18,6.05),(19,6.21),(20,6.37),(80,6.55) | ([7](#_ENREF_7), [8](#_ENREF_8), [13](#_ENREF_13)) |
| ${{FMI}_{Mal}}^{Ref}$ | (Age in Year, Reference FMI in Kg/M^2^) (0,1.6),(0.25,5.1),(0.5,5.03),(0.75,4.49),(1,4.42),(1.5,4.15),(2,4.04),(3,3.54),(4,3.21),(5,2.84),(6,2.65),(7,2.45),(8,2.54),(9,2.98),(10,3.37),(11,3.52),(12,3.4),(13,3.18),(14,3.02),(15,2.9),(16,2.84),(17,2.9),(18,3.14),(19,3.55),(20,4.01),(80,5.31) |  |
| ${{CL}_{Fem}}^{Ref}$ | (Age in Year, Reference Fractional Cellularity) (0,0.85),(1,0.894),(2,0.928),(3,0.947),(4,0.958),(5,0.966),(6,0.974),(7,0.98),(8,0.988),(9,0.997),(10,1), (20,1), (80,0.895) | ([5](#_ENREF_5)) |
| ${{CL}_{Mal}}^{Ref}$ | (Age in Year, Reference Fractional Cellularity) (0,0.8),(1,0.83),(2,0.871),(3,0.897),(4,0.918),(5,0.934),(6,0.946),(7,0.957),(8,0.968),(9,0.979),(10,0.99),(11,1),(20,1),(80,0.825) |  |
| $\sum_{i=1}^{4} \frac{{M_{i}}^{Ref}}{{BW}^{Ref}}$ | (0,0.152),(1,0.145),(2,0.132),(3,0.124),(4,0.119),(5,0.106),(6,0.0984),(7,0.0917),(8,0.0833),(9,0.0803),(10,0.077),(11,0.0732),(12,0.0707),(13,0.0663),(14,0.062),(15,0.0592),(16,0.0578),(17,0.0566),(18,0.0573),(20,0.0543) | ([4](#_ENREF_4)) |
| $\sum_{i=1}^{4} \gamma_{i}\frac{{M_{i}}^{Ref}}{{BW}^{Ref}}$ | $Max(31.4+189.9e^{-0.107.age},59.05)$ | ([5](#_ENREF_5)) |
| ${{PAL}_{Fem}}^{Ref}$ | (Age in Year, Reference PAL) (0,1.3),(1.5,1.42),(2.5,1.42),(3.5,1.44),(4.5,1.49),(5.5,1.53),(6.5,1.56),(7.5,1.6),(8.5,1.63),(9.5,1.66),(10.5,1.71),(11.5,1.74),(12.5,1.76),(13.5,1.76),(14.5,1.75),(15.5,1.73),(16.5,1.73),(17.5,1.72),(20,1.62),(30,1.48),(40,1.41),(50,1.36),(60,1.32),(70,1.28),(80,1.25) | ([13](#_ENREF_13), [14](#_ENREF_14)) |
| ${{PAL}_{Mal}}^{Ref}$ | (Age in Year, Reference PAL) (0,1.3),(1.5,1.43),(2.5,1.45),(3.5,1.44),(4.5,1.49),(5.5,1.53),(6.5,1.57),(7.5,1.6),(8.5,1.63),(9.5,1.66),(10.5,1.71),(11.5,1.75),(12.5,1.79),(13.5,1.82),(14.5,1.84),(15.5,1.84),(16.5,1.84),(17.5,1.83),(20,1.75),(30,1.55),(40,1.45),(50,1.38),(60,1.33),(70,1.3),(80,1.27) |  |

Simulations use linear interpolation between data-points reported in the table.

Table 5- Model parameters used in the reported simulations.

| **Parameter** | **Value** | **Unit** | **Comment** |
| --- | --- | --- | --- |
| *d* | 0.02 | Year | Growth projection horizon |
| τ | 30 | Days | Time to consume or adjust body mass reserves |
| $s_{H}$ | 8 | Dimensionless | Slope of height growth rate reaction to relative BMI |
| $v_{H}$ | 4 | Dimensionless | Maximum catch up growth rate relative to normal height growth for age ([11](#_ENREF_11)) |
| $c$ | 10.4 | Kg | Based on Forbes partitioning equation ([3](#_ENREF_3)) |
| ${\rho_{L}}^{Adult}$ | 5000 | KJ/Kg | Maximum energy density of lean mass ([1](#_ENREF_1)) |
| ${\rho_{L}}^{Base}$ | 3514 | KJ/Kg | Minimum energy density of lean mass ([1](#_ENREF_1)) |
| $\alpha_{\rho}$ | 18 | KJ/Kg^2^ | Slope of lean mass energy density with respect to lean mass ([1](#_ENREF_1)) |
| $\rho_{F}$ | 39500 | KJ/Kg | Energy density of fat mass ([15](#_ENREF_15)) |
| $\eta_{L}$ | 960 | KJ/Kg | Energy need for turnover of lean mass ([15](#_ENREF_15)) |
| $\eta_{F}$ | 750 | KJ/Kg | Energy need for turnover of fat mass ([15](#_ENREF_15)) |
| $f_{FM}$ | 0.1 | Dimensionless | Fat mass fraction of active metabolic organs ([5](#_ENREF_5)) |
| $\gamma_{\Delta L}$ | 92 | KJ/Kg/Day | Energy requirements of active organs ([15](#_ENREF_15)) |
| $\gamma_{5}$ | 54 | KJ/Kg/Day | Energy requirement of structural muscle and other residual lean tissue ([5](#_ENREF_5), [16](#_ENREF_16)) |
| $\beta$ | 0.1 | Dimensionless | Thermic cost of eating ([15](#_ENREF_15)) |
| *sb* | 0.5 | Dimensionless | Weight of CDC based projection on desired weight |
| $\beta_{T}$ | 0.14 | Dimensionless | Adaptive thermogenesis effect ([15](#_ENREF_15)) |
| $\gamma_{F}$ | 13 | KJ/Kg/Day | Energy requirements for unit fat mass ([15](#_ENREF_15)) |

**References**

1. Hall KD, Butte NF, Swinburn BA, Chow CC. Quantifying the Dynamics of Childhood Growth and Obesity. Lancet Diabetes and Endocrinology 2013;1(2):97-105.

2. Forbes GB. Lean body mass-body fat interrelationships in humans. Nutrition reviews 1987;45(8):225-31.

3. Hall KD. Body fat and fat-free mass inter-relationships: Forbes's theory revisited. The British journal of nutrition 2007;97(6):1059-63. doi: 10.1017/S0007114507691946.

4. Altman PL, Dittmer DS. Growth including reproduction and morphological development. Washington,: Federation of American Societies for Experimental Biology, 1962.

5. Wang ZM. High ratio of resting energy expenditure to body mass in childhood and adolescence: A mechanistic model. Am J Hum Biol 2012;24(4):460-7. doi: Doi 10.1002/Ajhb.22246.

6. Hall KD. Predicting metabolic adaptation, body weight change, and energy intake in humans. Am J Physiol Endocrinol Metab 2010;298(3):E449-66. doi: ajpendo.00559.2009 [pii]

10.1152/ajpendo.00559.2009.

7. Butte NF, Hopkinson JM, Wong WW, Smith EO, Ellis KJ. Body composition during the first 2 years of life: an updated reference. Pediatric research 2000;47(5):578-85.

8. Wells JC, Williams JE, Chomtho S, et al. Body-composition reference data for simple and reference techniques and a 4-component model: a new UK reference child. Am J Clin Nutr 2012;96(6):1316-26. doi: 10.3945/ajcn.112.036970.

9. Fomon SJ, Haschke F, Ziegler EE, Nelson SE. Body-Composition of Reference Children from Birth to Age 10 Years. American Journal of Clinical Nutrition 1982;35(5):1169-75.

10. Boersma B, Wit JM. Catch-up growth. Endocrine reviews 1997;18(5):646-61.

11. Walker SP, Golden MH. Growth in length of children recovering from severe malnutrition. European journal of clinical nutrition 1988;42(5):395-404.

12. Kuczmarski RJ, Ogden CL, Grummer-Strawn LM, et al. CDC growth charts: United States. Adv Data 2000(314):1-27.

13. CDC. Internet: <http://www.cdc.gov/nchs/nhanes.htm>.

14. Torun B. Energy requirements of children and adolescents. Public health nutrition 2005;8(7A):968-93. doi: Doi 10.1079/Phn2005791.

15. Hall KD, Sacks G, Chandramohan D, et al. Quantification of the effect of energy imbalance on bodyweight. Lancet 2011;378(9793):826-37. doi: S0140-6736(11)60812-X [pii]

10.1016/S0140-6736(11)60812-X.

16. Wang Z, Heshka S, Heymsfield SB, Shen W, Gallagher D. A cellular-level approach to predicting resting energy expenditure across the adult years. Am J Clin Nutr 2005;81(4):799-806. doi: 81/4/799 [pii].

1. <http://vensim.com/allocation-by-priority-alloc-p/> [↑](#footnote-ref-1)
