# Supplementary material for: Human Growth and Body Weight Dynamics: An Integrative Systems Model
Source: PLoS One. 2014 Dec 5;9(12):e114609. doi: 10.1371/journal.pone.0114609 (PMC4257729; doi:10.1371/journal.pone.0114609)
Supplement: Appendix S2 — Comparison of different body composition equations. (DOCX) [file pone.0114609.s002.docx]

### B- Comparison of different body composition equations

Equations used for estimating FMI as a function of current or indicated BMI (equations S22 and S23) are critical in the current model as they drive partitioning of body weight into FM and FFM. The current functional structure for the estimating equations were chosen after comparing many alternative functional forms, e.g. by including Log(BMI) and 1/BMI as potential independent variables. The current form proved most accurate in predicting FMI values.

I also compared the functional form for FM predictions (as a function of BW) that results from the use of Forbes partitioning equations. Forbes equations are estimated using the regression functional form:

$FFM=a.Ln(FM)+b$ S31

To compare predictions from my equation with those resulting from the above structure, I estimated this form using NHANES data on FM and FFM for a sample of 4213 non-hispanic white adult subjects (approximately half male and half female). Note that focusing on only adult samples for this comparison is conservative as Forbes equation structure is only tested for adults. This estimation results in the following equations for male and female:

${FFM}_{Male}=21.71+12.67Ln(FM)$ S32

${FFM}_{Female}=-3.35+14.06Ln(FM)$ S33

I then solved the resulting regression equation to find FM as a function of BW(=FFM+FM). There is no analytical solution for this equation, yet numerical methods can be used to find the resulting estimates for Forbes equation structure, predicting FM as a function of BW. I compared these predictions with those from equations following the structure used in this paper, but for fair comparison, estimated using only the same data and no other variables:

${FMI}_{Male}=3.19-0.184BMI+0.0115{BMI}^{2}$ S34

${FMI}_{Female}=-3.8+0.474BMI+0.0022{BMI}^{2}$ S35

Once transformed to predict FM based on BW, as in the case of Forbes derivative, these equations performed better on mean absolute percentage error (37% vs. 47% for male; 14% vs. 15% for female) and r-squared (0.81 vs. 0.79 for male; 0.946 vs. 0.945 for female) in these prediction comparisons. This provided further confidence in the ability of the equations utilized in this paper, which follow the same functional structure, to capture the relationship between BMI and fat mass.

Note that the actual equations used in the model are more precise due to a few additions: 1) They include an age varying reference BMI and use the variations around this factor to estimate the effect of BMI on FMI. 2) They include the ethnicity factor as a separate variable. 3) They use data for both children (over 8) and adults (under 50) in estimating a single equation. As a result the model provides significantly better predictions of body composition compared to the simpler structure explained above.
