# Supplementary material for: Human Growth and Body Weight Dynamics: An Integrative Systems Model
Source: PLoS One. 2014 Dec 5;9(12):e114609. doi: 10.1371/journal.pone.0114609 (PMC4257729; doi:10.1371/journal.pone.0114609)
Supplement: Appendix S3 — Using the simulation model. Instructions for using the simulation model in Appendix S4. (DOCX) [file pone.0114609.s003.docx]

### C- Using the simulation model

The simulation model used in this study is available for independent analysis and inspection. The model is made available in two forms. First, it is available as a stand-alone package which can be opened by Vensim Model Reader (free software) and is funcational for most basic operations and analyses. This package is available as HumanGrowthDynamic_V36_4.vmf in the AppendixS4.zip. The Vensim™ simulation language is used to construct and analyze these models. Vensim packages can be easily opened, inspected, and simulated with the free Vensim Model Reader software available for download from: <http://vensim.com/freedownload.html>.

Second, the FullAnalysis folder (included in the AppendixS4.zip) includes all of the model files, data files, and commands required for fully replicating the analysis reported in the paper. Opening and running these files requires the Vensim DSS which is not free, and some familiarity with this simulation environment. However, once you have unzipped the FullAnalysis folder, you can go inside Vensim DSS, open the FullAnalysis.cmd and click on “Run Commands…” from the File menu. That will replicate all of the simulations conducted for the analysis reported in the paper and will store the relevant outcomes in tab files which you can use for further processing. Those familiar with this environment can also inspect the command file and the auxiliary files to better understand the changes made for each step of the analysis. Conducting the various analysis reported in the paper required several different versions of the model which all include the same basic structure but vary in the number of subscripts and some auxiliary variables used for collecting additional information or connecting the models with data, and conducting optimizations. The additional instructions below are useful for those not familiar with Vensim so that they can easily conduct analysis using the package (.vmf) file provided and the free Vensim model reader.

#### Opening, inspecting, and running the models

You can open the model (.vmf file) and analyze it using the simulation environment provided by the Vensim Model Reader or professional editions of the software. The model comes in 3 views, among which you can navigate with page-up or page-down keys. Most relevant variables and graphs are included in the second, “Control Panel” view where most users can concentrate their attention on. Complete model is visible under the third view, “Full Model”. The model is formulated based on the equations discussed in Section A and most equations include further comments explaining their logic.

The model includes six simulated individuals, to facilitate parallel comparison. They are implemented as subscripts (i.e. copies of the same model indexed by the subscript “People”. The subscript control box, accessible by clicking on “Subscripts” button
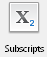
, allows one to select any subset of the people you want to focus on, while all 6 are being simulated in the background at all times.

The control panel view includes four sets of variables and parameters to facilitate interactions with the model, as well as two custom graphs and a reports (See below). The variables are organized under four headings:

**Demographics and Initialization**- Use these parameters to initialize your simulated individuals; for example you can set the initial weight (BMI) of an individual by changing “InitBMIFac”.

**Reference Curves**- These variables specify the reference curves used for BMI, FMI, Height, PAL, and components of energy expenditure. You may change physical activity levels here, or customize the model to a new population using different reference curves.

**Changing Energy Intake**- This section includes the inputs into the energy intake equation in the model. The model includes the capability to simulate an individual in endogenous dynamic equilibrium (where energy intake equals total energy expenditure and energy for reference growth), one who consumes proportionally more/less than this trajectory, or a completely exogenous intake.

**Simulation Control**- Parameters controlling the simulation experiment, including initial and final time, simulation time step, and frequency of saving data for output processing.


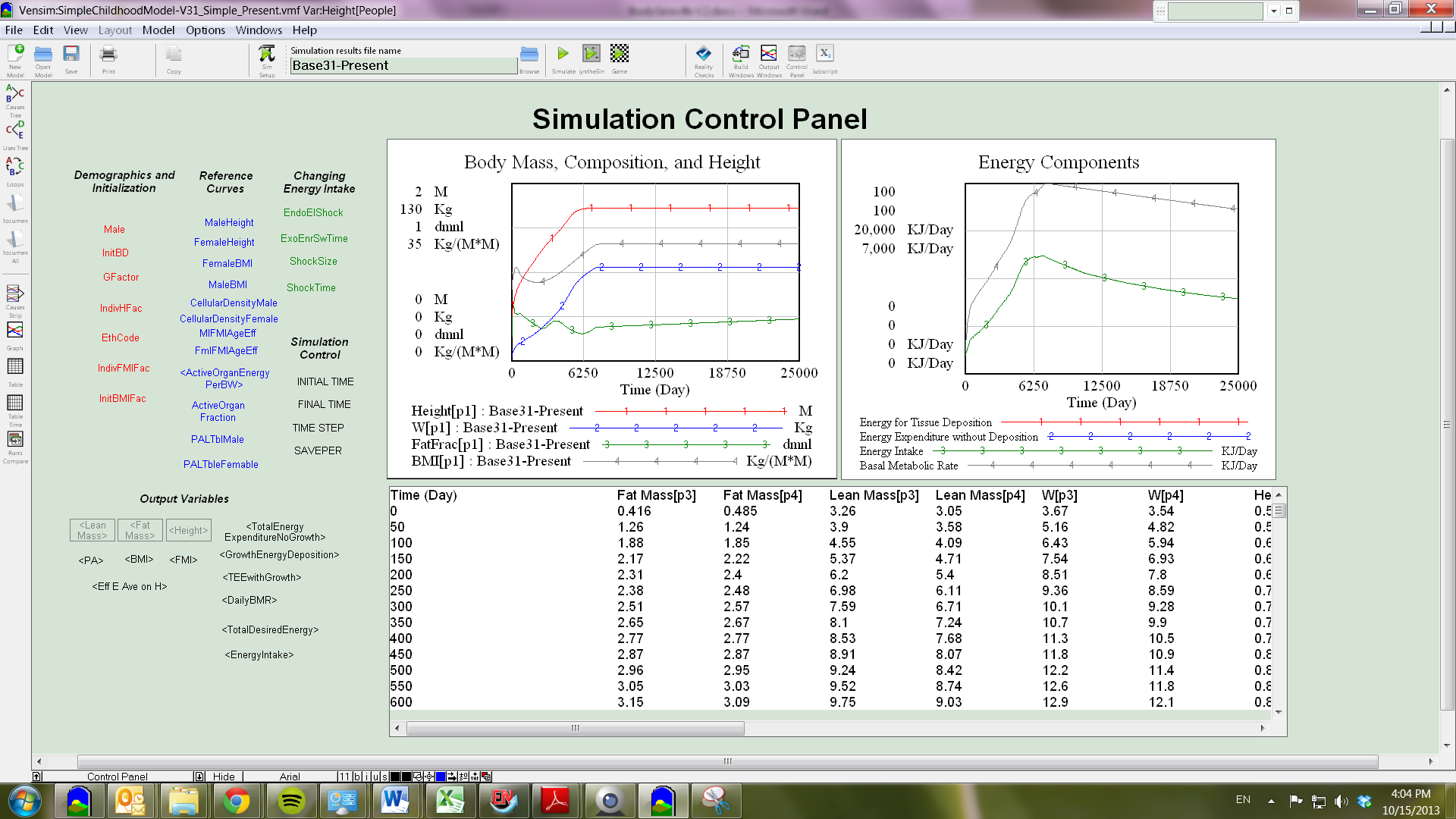


You can view the equation for any variable on any view by selecting that variable and clicking the “Document” button in the left toolbar,
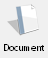
. You can follow the procedure below for simulating and analyzing the model behavior:

- First choose a name for your simulation and enter it in the field for simulation name in the middle of the top toolbar:
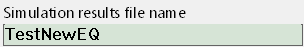

- Click on the simulation setup button,
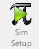
, to the left of this name.
- Now change the parameters of the model as desired. The current values of the parameters are shown if you click on each parameter. The attached models include the base values used for reported simulations in the paper.
- Simulate the model by clicking the Simulate button in the top toolbar:
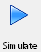
.

This procedure is applicable for simulating the base run on any model.

#### Examining model behavior

The graphs and the numerical report on the control panel would provide a good overview of the model behavior. You can also use the tools in the left toolbar to see the behavior of different variables. Select a variable by clicking on it and then click on the desired tool (graph or table). A graph or table of the variable of interest will be shown. You can view all model variables in this way.

Note that, you cannot edit a model in the Vensim Model Reader. For that purpose you will need the Professional or DSS versions of Vensim for editing this model (due to use of subscripts and allocation function this model can not be simulated in the free Vensim PLE).
